# Clinicopathological Characteristics and Survival Outcomes of Patients with Buccal Squamous Cell Carcinoma: Results of a Multi-Institutional Study

**DOI:** 10.3390/medicina57121361

**Published:** 2021-12-13

**Authors:** Shogo Shinohara, Masahiro Kikuchi, Hiroyuki Harada, Kiyomi Hamaguchi, Ryo Asato, Hisanobu Tamaki, Masanobu Mizuta, Ryusuke Hori, Tsuyoshi Kojima, Keigo Honda, Takashi Tsujimura, Yohei Kumabe, Kazuyuki Ichimaru, Yoshiharu Kitani, Koji Ushiro, Koichi Omori

**Affiliations:** 1 Department of Otolaryngology—Head & Neck Surgery, Kobe City Medical Center General Hospital, Kobe 650-0047, Japan; kiyomi_hamaguchi@kcho.jp; 2 Department of Otolaryngology—Head & Neck Surgery, Graduate School of Medicine, Kyoto University, Kyoto 606-8507, Japan; m_kikuchi@ent.kuhp.kyoto-u.ac.jp (M.K.); omori@ent.kuhp.kyoto-u.ac.jp (K.O.); 3 Department of Otolaryngology—Head & Neck Surgery, Medical Research Institute, Kitano Hospital, Osaka 530-8480, Japan; h_harada@ent.kuhp.kyoto-u.ac.jp; 4 Department of Otolaryngology—Head & Neck Surgery, National Hospital Organization Kyoto Medical Center, Kyoto 612-8555, Japan; asato@ent.kuhp.kyoto-u.ac.jp; 5 Department of Otolaryngology—Head & Neck Surgery, Kurashiki Central Hospital, Okayama 710-8602, Japan; ht14818@kchnet.or.jp (H.T.); m_mizuta@ent.kuhp.kyoto-u.ac.jp (M.M.); 6 Department of Otolaryngology, Tenri Hospital, Nara 632-0015, Japan; ryusuke.hori@fujita-hu.ac.jp (R.H.); t_kojima@ent.kuhp.kyoto-u.ac.jp (T.K.); 7 Department of Otolaryngology, Japanese Red Cross Wakayama Medical Center, Wakayama 640-8558, Japan; kegohonda@gmail.com; 8 Department of Otolaryngology, Head and Neck Surgery, Japanese Red Cross Otsu Hospital, Otsu 520-0046, Japan; ttakashi502@gmail.com; 9 Department of Otolaryngology—Head & Neck Surgery, Hyogo Prefectural Amagasaki General Medical Center, Amagasaki 660-8550, Japan; pa45328@gc4.so-net.ne.jp; 10 Department of Otolaryngology—Head & Neck Surgery, Kokura Memorial Hospital, Fukuoka 802-8555, Japan; ichimaru-k@kokurakinen.or.jp; 11 Department of Otorhinolaryngology—Head & Neck Surgery, Shizuoka General Hospital, Shizuoka 420-8527, Japan; y_kitani@ent.kuhp.kyoto-u.ac.jp; 12 Department of Otorhinolaryngology—Head & Neck Surgery, Shiga General Hospital, Moriyama 524-8524, Japan; koji.ushiro@gmail.com

**Keywords:** buccal cancer, oral cancer, survival outcome, Japan

## Abstract

*Background and Objectives*: To investigate clinicopathological characteristics and survival outcomes of patients with buccal cancer in Japan. *Materials and Methods*: This study was conducted using a database of 1055 patients with oral cancers treated between 2010 and 2017 at 12 institutions in Japan. Ninety-two patients (8.7%) with primary buccal cancer were extracted and clinicopathological characteristics and survival outcomes were compared between patients with buccal cancers and patients with other oral cancers. *Results*: Ages were significantly higher in the patients with buccal cancer (73 years old vs. 69 years old). Buccal cancer had less advanced cT stage and cN stage than other oral cancers. Overall 5-year survival (OS) was 80.6%, and recurrence-free 5-year survival (RFS) of buccal cancers was 67.8%, and there were no significant differences in survival compared with other oral cancers in terms OS or RFS (5y-OS: 82.5%, 5y-RFS: 74.4%). However, patients with stage IV buccal cancer showed poorer prognosis in terms of OS and RFS compared with the same stage patients with other oral cancer. Advanced T stage was the only factor independently associated with both OS and RFS of patients with buccal cancer in this study. *Conclusions*: Postoperative radiotherapy or chemoradiotherapy should be considered to improve survival outcome of buccal cancer patients, especially for the patients with advanced primary site disease or a higher cancer stage.

## 1. Introduction

Buccal cancer is a type of oral cancer. This cancer is reported to occupy a minor portion of oral cancer, accounting for less than 10% of cases in Untied States and European countries [1,2]. Indeed, according to ICD-10-CM (International Statistical Classification of Diseases and Related Health Problems, Clinical Modification) codes, buccal cancer is categorized into C06: malignant neoplasm of other and unspecified parts of mouth, while palate cancer and lip cancer have their own ICD-10 codes, C04 and C00, respectively.

For the head and neck oncologist, surgical treatment of buccal cancer is sometimes challenging because of its anatomical characteristics. The buccinator muscle, which is the main structure and functional component of the cheek, serves as an anatomic barrier against infection or invasion of malignancy arising on the buccal mucosa. However, once cancer penetrates the buccinator muscle, it may spread through the buccal space to neighboring organs, such as maxilla, mandible, and infratemporal fossa, and it easily penetrates external skin. The invasion to infratemporal fossa may cause trismus, which makes it difficult for conducting observation and performing oral surgical treatment. Moreover, reconstruction after resection of advanced buccal cancer is sometimes puzzling when a surgical defect involves mandible, maxilla, and external skin of the cheek.

In Japan, buccal cancer has been treated by dental surgeons licensed to practice dentistry and by head and neck surgeons licensed to practice medicine. For these reasons, there have been few papers with a large sample size reporting clinicopathological characteristics and survival outcomes of patients with buccal cancer in Japan. In this study, we utilized a multi-institutional database in Japan and investigated clinicopathological characteristics and survival outcomes of patients with buccal cancer in this country.

## 2. Materials and Methods

### 2.1. Patient’s Data Source

**Figure 1:**
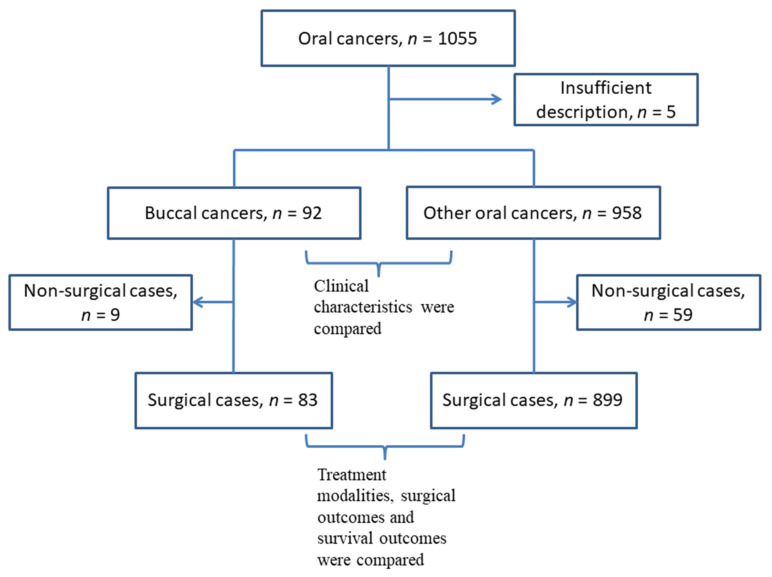
A flow chart of the inclusion and exclusion process of the patients.

This study was performed in 12 institutes associated with Kyoto University and its Affiliated Hospitals—Head and Neck Oncology Group (Kyoto–HNOG) in Japan. Clinical data for patients with oral cancer treated between March 2010 and February 2017 were retrospectively extracted from medical charts. The patients’ data were anonymized and utilized for another previous research study examining the impact of lingual lymph node metastases on patients’ survival [3]. In this data source, one thousand and fifty-five patients were enrolled, and the location of the primary tumor was categorized into 7 subsites: tongue, floor of mouth, hard palate, upper gingiva and lower gingiva, oral lip, and buccal mucosa. Five cases that were not categorized using TNM classification 8th version were excluded from the study. Subsequently, patients with buccal primary tumors were separated, and the data were dichotomized into 2 groups: 92 buccal cancers (8.8%) and 958 other oral cancers (91.2%) (Figure 1). This study was approved by the institutional review board of each participating institution in collecting patients’ data and was led by the Kobe City Medical Center General Hospital Review Board (ethics code: Zn191105). Informed consent was waved owing to the retrospective nature of this study.

### 2.2. Patient’s Demographics and Clinical Characteristics

To understand the demographic and clinical characteristics of the patients with buccal cancer, the individual variables were listed and compared with the patients with other oral cancers. The valuables were age at diagnosis, gender, smoking/drinking habit, TNM classification, treatment modality, postoperative therapy, postoperative positive nodes, and pathological surgical margin. In this series of the study, the latest version of TNM classification, the 8th AJCC/UICC classification, was utilized. We had asked each institute to perform re-staging of all cases according to the 8th AJCC/UICC classification using radiological examination and/or the medical chart of the patients. Cases with unknown TNM stages were excluded from the data, as mentioned in Section 2.1.

### 2.3. Survival Analysis

For analyses of the survival outcome of the patients who were treated with curative intent, the patients who were treated with palliative radiotherapy, chemotherapy, or best supportive care only were omitted in this step (Figure 1). In order to investigate the factors that affect the overall survival or the recurrence-free survival, clinicopathological covariates were dichotomized for statistical evaluation: age (dichotomized by median value), gender (male vs. female), cT stage (cTis/T1/T2 vs. cT3/T4), cN stage (cN0 vs. cN1–3), clinical stage (I–II vs. III–IV), pN stage (pN0 vs. pN1–3), neck dissection at the initial treatment (yes vs. no), postoperative treatment, such as postoperative radiotherapy, postoperative chemoradiotherapy, and/or adjuvant chemotherapy (yes vs. no), surgical margin (negative vs. positive), extracapsular extension of metastatic lymph nodes (positive vs. negative/no positive nodes). The overall survival (OS) and recurrence-free survival (RFS) were estimated by the Kaplan–Meier method, and the differences between groups were assessed using the log-rank test. Cox multivariate regression analysis was used to investigate the factors that independently affected the patients’ survival in terms of OS or RFS.

### 2.4. Statistics Analysis

The Mann–Whitney U test was used to compare variables, such as the patients’ age between groups. The association for the categorical variables was compared using the chi-square test. Survival outcomes, overall survival (OS), and recurrence-free survival (RFS), were estimated using the Kaplan–Meier method, and groups were compared using the log-rank test. Cox proportional hazard regression models were used to determine the relationship between patients’ clinical characteristics, surgical outcomes, and OS or RFS. The analyses were performed with EZR on R commander, version 1.42, and a *p* value of <0.05 was considered to be significant.

## 3. Results

### 3.1. The Difference in Patient Characteristics between Buccal Cancer Patients and Other Oral Cancer Patients

The clinicopathological and treatment characteristics of 92 buccal cancer patients in this study are listed in Table 1. Pathologically, squamous cell carcinomas were dominant (88 cases), followed by spindle cell carcinomas (3 cases) and verrucous carcinoma (1 case); both were recognized as subtypes of squamous cell carcinoma. Males were dominant and age ranged from 47 to 94 years old, with a median value of 73. Almost one-third of the patients were smokers, and 40% of patients were habitual alcohol consumers. More than 70% of patients (70/92) had early stage of primary lesions, and clinical positive nodes were identified in 28% (26/92) of the patients. One patient had distant metastasis at the first visit to the institution. A total of 83 (90%) patients underwent surgery with a curative intent, but 9 patients were treated with a palliative intent because of advanced stage of the disease, poor general conditions, or patients refusal of surgical procedures.

The clinicopathological and treatment characteristics of 958 other oral cancer patients in this study are also listed in Table 1. Pathologically, squamous cell carcinomas were also dominant (945 cases), followed by verrucous carcinomas (5 cases), spindle cell carcinomas (4 cases), adenosquamous carcinomas (3 cases), and undifferentiated carcinoma (1 case). Ages were significantly lower in the patients with other oral cancers ranging from 21 to 98 years old, with a median value of 69. The distributions of gender were almost identical. The rates of smoking tobacco and drinking alcohol were similar in both groups. The patients with other oral cancers had significantly higher clinical T stage and clinical N stage than the patients with buccal cancers (*p* = 0.01 and *p* < 0.01, respectively); however, the difference in clinical stage was not significant (*p* = 0.20). In the other oral cancer group, 899 patients (94%) underwent surgery with a curative intent, but 59 patients (6%) were treated with a palliative intent.

The characteristics of surgical cases in both groups are compared in Table 2. A major portion of the primary site (93%) was resected orally in the buccal cancer group, and external resection, such as pull through resection for tongue cancers, was selected significantly more often in the other oral cancer group (18%; *p* = 0.01). Neck dissections were performed in significantly more patients in the other oral cancer group (*p* < 0.01). The free frap technique was utilized in 10 patients (12%) in buccal cancer and 132 patients (15%) in other oral cancers for the reconstruction of the primary site. Artificial materials, such as a polyglycolic acid (PGA) sheet, a scaffold of regenerating tissue, fixed with fibrin glue were used for covering the defect of the primary site in more than half of cases in both groups. In the buccal cancer group, cancer-positive lymph nodes were detected in 19 patients (23%) among the 30 patients who received neck dissection, and 4 (5%) of them were proved to have extracapsular extension pathologically. In the other oral cancer group, cancer-positive lymph nodes were detected in 214 patients (24%) among the 479 patients who received neck dissection and 58 (6%) of them were proved to have extracapsular extension pathologically. The pathological surgical margins were reported to be positive in 4 patients (5%) in the buccal cancer group and 71 patients (8%) in the other oral cancer group. Postoperative radiotherapy, concurrent chemoradiotherapy, or adjuvant chemotherapy were performed in 18% and 24% of high-risk buccal and other oral cancer groups, respectively.

### 3.2. Survival Outcomes of Buccal Cancer Patients

**Figure 2:**
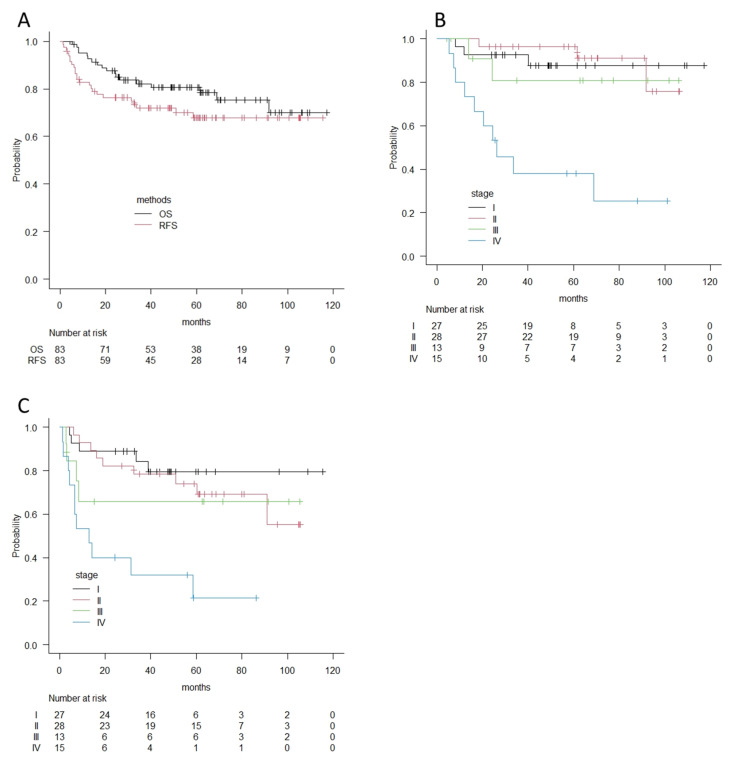
(**A**) Overall survival curves and recurrence-free survival curves for 83 patients with buccal cancer according to the Kaplan–Meier method. (**B**) Overall survival curves by clinical stages for patients with buccal cancer according to the Kaplan–Meier method. (**C**) Recurrence-free survival curves by clinical stages for patients with buccal cancer according to the Kaplan–Meier method. OS; Overall survival, RFS; Recurrence-free survival.

For analyses of the survival outcome of buccal cancers, 9 patients treated with palliative therapy were omitted, and 83 patients who received surgery with curative intent were selected. For these 83 patients, the median follow-up period was 56 months. A total of 14 patients died of the disease during follow-up, whereas 7 patients died of other causes, including 3 cancer deaths in other primary sites. A total of 24 patients experienced recurrence during follow-up: 14 in primary sites, 11 in cervical lymph nodes, and 8 in distant organs. Among them, 7 patients were surgically salvaged, and 6 patients were alive with no evidence of disease during follow-up. The overall 2-year and 5-year survival rates were 87.6% and 80.6%, and the 2-year and 5-year recurrence-free survival rates were 76.4% and 67.8% (Figure 2A). According to clinical stage, overall survival curves and recurrence-free survival curves were similar in stage I, II, and III patients, whereas patients with stage IV disease had much poorer outcomes—around 38% in 5-year OS and 21% in 5-year RFS (Figure 2B,C).

Univariate analyses using the log-rank test were performed using the dichotomized covariates in Table 3. Advanced T stage (T3–4), positive clinical N stage, and advanced clinical stage (III–IV) significantly affected both overall survival and recurrence-free survival. A pathological positive node only affected recurrence-free survival. Postoperative treatment was significantly associated with recurrence-free survival, which was probably because these patients were considered to be high-risk.

In multivariate analyses, advanced T stage (T3–4) was an independent significant risk for patients’ overall survival and recurrence-free survival, with a hazard ratio 4.92 (95% confidence interval: 1.52–15.9, *p* < 0.01) and 3.08 (95% confidence interval: 1.00–9.50, *p* = 0.04). For recurrence-free survival, neck dissection was independently associated with worse survival outcome, with a hazard ratio 0.12 (95% confidence interval: 0.02–0.72, *p* = 0.02) (Table 4).

### 3.3. The Comparison of Survival Outcomes between Buccal Cancer Patients and Other Oral Cancer Patients

**Figure 3:**
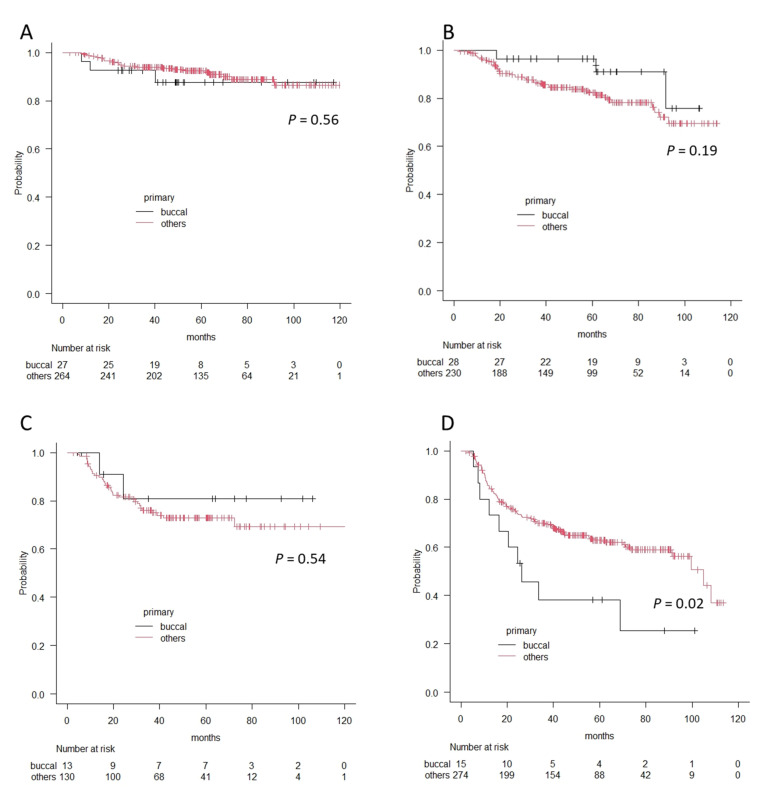
The differences in overall survival curves between patients with buccal cancer and patients with other oral cancers according to the Kaplan–Meier method. (**A**) Stage I, (**B**) Stage II, (**C**) Stage III, and (**D**) Stage IV. A significant difference is observed in patients with Stage IV.

**Figure 4:**
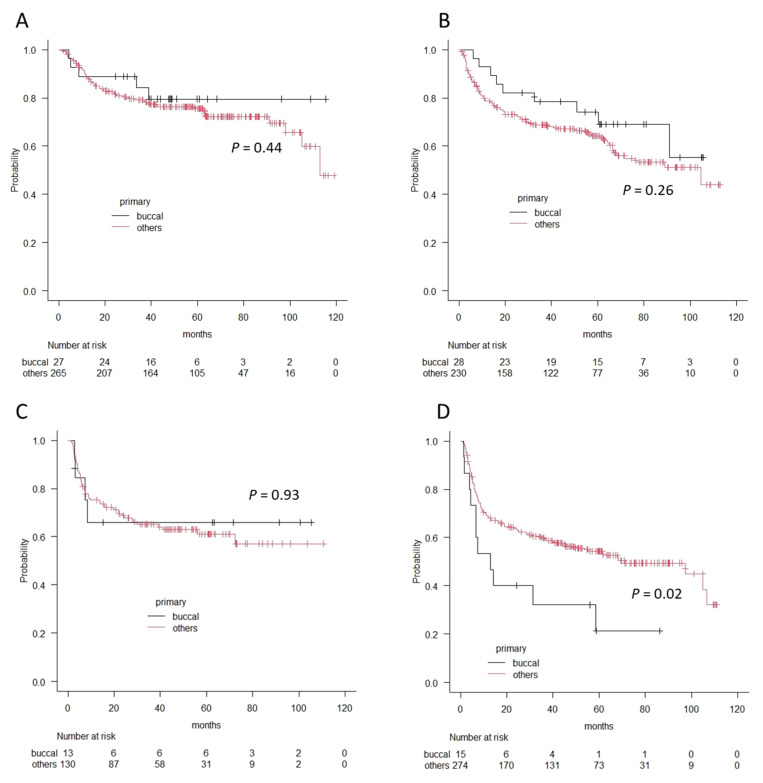
The differences in recurrence-free survival curves between patients with buccal cancer and patients with other oral cancers according to the Kaplan–Meier method. (**A**) Stage I, (**B**) Stage II, (**C**) Stage III, and (**D**) Stage IV. A significant difference is observed in patients with Stage IV.

Finally, we compared survival outcomes between the patients with buccal cancer and those with other oral cancers using the Kaplan–Meier method and log-rank test. Overall 5-year survival rates were 80.6% and 82.5%, and recurrence-free 5-year survival rates were 67.8% and 74.4% for buccal cancer and other oral cancers, respectively. There were no significant differences in each survival outcome (*p* = 0.8 in OS and *p* = 0.97 in RFS). However, in accordance with clinical stages, the patients with stage IV buccal cancer showed significantly worse prognoses in terms of overall survival and recurrence-free survival (OS; 38.1% vs. 62.9%, *p* = 0.02, RFS; 21.3% vs. 54.3%, *p* = 0.02), whereas there was no difference in Stage I, II, and II in terms of OS or RFS (Figure 3 and Figure 4).

## 4. Discussion

Buccal cancer is regarded to be an uncommon neoplasm of the oral cavity in North America and Western Europe, reported to account for approximately 10% of oral cavity cancers [1,2]. The incidence of buccal cancer varies by country due to the habits of each nation. In India, there is a habit of chewing tobacco and placing a quid containing tobacco in the gingivobuccal sulcus, and the incidence of buccal cancer was reported to be up to 41% of oral cavity cancers and 10% of all head and neck cancers [4]. In Taiwan, there is a habit of chewing betel quid, consisting of betel leaf, areca nut, and lime, and the incidence of buccal cancer was reported to be 37% of oral cavity cancers [5]. These materials are related to carcinogenesis of the buccal mucosa. In the present study, the incidence of buccal cancer among all oral cavity cancers was 8.8%, which may represent the incidence of buccal cancer in Japan because the data analyzed in this study were gathered from multiple institutes around Japan. This incidence seems to be reasonable because we do not have a habit of chewing tobacco, and the incidence is similar to the incidence in the United States, 7.4% using the Surveillance, Epidemiology, and End Results database (SEER database) from 2004 to 2009 [1].

In the present study, 25% of the cancer patients had advanced stage of the primary site (T3/T4), and 28% of the patients had clinically positive nodes. As for clinical stage, 39% of the patients had advanced stage (III/IV). In the previous reports, the rate of advanced T stage ranged widely, from 21% to 81% [2,4,6,7,8,9]. Pradhan et al. reported in 1989 that 81% of the patients had T3/T4 tumors, which were treated at the Tata Memorial Hospital in India [4], whereas 21% out of 113 patients in an institute in Germany had advanced T stage, which was reported by Segheb et al. in 2017 [9]. On the other hand, the rate of clinically positive nodes ranged from 22% to 45% in the literature [2,4,6,7,8,9,10]. The differences in the rate of advanced T stage and clinically positive nodes were thought to depend on the timing and country in which the investigators examined, as well as on the scale and the role of the institute they belonged to.

In the present study, the rate of T1 cancer was almost similar in buccal cancer patients (28/92, 30%) and other oral cancers (283/958, 30%), which did not agree with the past literature. Shaw et al. reported that buccal cancers have a significantly lower rate of T1 tumors compared with other sites of oral cancers (16% vs. 28%) [11]. Camilon et al. investigated patient demographics in the United States using the SEER database, extracting 824 patients with buccal cancer from 11,134 patients with oral cancer and reported that buccal cancer had significantly fewer stage I tumors than the other oral cancers (27% vs. 36%) [1]. The patients with early buccal cancer are thought to have fewer symptoms than those with other oral cavity cancers and may notice the disease in a more advanced stage. The reason why the patients with buccal cancer in our study had a significantly lower clinical T stage than those with the other oral cavity cancers was uncertain; however, it might be attributed to the prevalence and intensity of dental care in Japan. Patients with early T stage buccal carcinoma without symptoms were often referred to us by their dentists. Camilon et al. also reported that buccal cancer patients presented at a significantly higher age than the other oral cancer patients, which agreed with the present study [1]. However, the mean age in this study, 73 years old, was much older than one in Camilon’s report, which was 67 years old.

Buccal cancer had been thought to have a poor prognosis due to its invasive tumor behavior and high incidence of locoregional recurrence [2,10,12]. The difficulty in obtaining negative surgical margins was responsible for the high incidence of locoregional recurrence [2,10], whereas patients in whom negative margins were achieved still marked a high incidence of local recurrence—52% in the literature [6]. In the present study, a negative margin was achieved in 95% of the patients, and local recurrence was observed in 14 out of 83 surgically treated patients (17%) during the observation period. The lower rate of positive surgical margin than previous reports was supposed to be attributed to routine use of an intraoperative frozen section. Sieczka et al. reported that 31% of T1 and T2 tumors and 20% of T3 and T4 lesions were resected with positive margins [6]. Advanced T stage was a factor independently associated with both overall survival and recurrence-free survival in this study, and this fact was also reported by Lin et al., who examined their 121 cases of buccal cancers [12]. These facts suggest that local control is the most important key to patients’ survival outcome. Postoperative radiotherapy or chemoradiotherapy would contribute to the improvement of patients’ survival, especially for the patients with advanced primary site disease or a higher cancer stage. The patients with stage IV disease had worse survival outcomes in terms of OS and RFS than those with the other oral cancers in the present study. One possible reason for this fact was that the rate of cT4b patients was higher in the buccal cancer group among all cT4 patients than in the other oral cancer group. Buccal cancer patients with cT4b primary site occupied 46% (6 patients) in cT4 patients (13 patients), while other oral cancer patients with cT4b primary sites occupied 5% (12 patients) in cT4 patients (221 patients) (Table 1). Buccal cancer may easily extend to the masticator space, which is currently defined as cT4b in the eighth TNM/AJCC classification.

Just like other oral cavity cancers, such as cancer of the tongue, the patients with buccal cancer often have occult cervical lymph node metastases in the cN0 neck. Hoda et al. reported that 145 out of 254 patients (57%) with buccal cancer who were treated surgically showed pathologically proven metastasis in the neck, out of which there were 56 patients showing occult metastasis [13]. Even in the early T stage of buccal cancer, cT2N0 tumors were demonstrated to be up to 10% of occult metastases in the literature [8], and the elective neck dissection (END) to cN0 neck was reported to improve cervical control rate [8] or disease specific survival [14]. In the present study, neck dissections were performed in 30 patients, out of which 11 patients received neck dissection as END. Pathological positive nodes were seen in 19 cases (63%) in all cases that received neck dissection, and 2 cases (18%) proved to have pathological positive nodes among 11 patients who were categorized into cN0 with preoperative work-up and who received END.

Finally, we discuss survival outcomes.

In this study, 2-year/5-year OS was 87.6%/80.6% and 2-year/5-year RFS was 76.4%/67.8%. In the previous literature, 5-year OS ranged widely, from 34% to 80% [1,9,12,14]. The report with the largest number of cases was written by Camilon et al. in 2014 [1]. They examined 825 cases between 2004 and 2009 using the SEER database, which covered approximately 28% of the population of the United States. They reported that the 2-year OS and the 5-year OS were 61% and 44%, respectively. Apparently, the survival outcome of our study seemed to be better than these results, but we analyzed survival outcome just for the patients who had been treated with curative intent. A total of 9 out of 92 patients treated with palliative therapy were omitted from the survival analyses in this study.

We recognize the limitation of this study. First, this study had a retrospective nature, and we had not determined the survey sample size a priori. The results of statistical analyses that did not reach to a significant difference might be due to the lack of a larger sample. Second, this study was a multi-institutional study, and the treatment strategy differed by institutes. A patient who received END might have been observed without END in another institute. Third, we did not have enough data on survival outcome for the patients who received palliative treatment. This made it difficult for us to compare survival data with previous reports.

## 5. Conclusions

Treating patients with buccal cancer, local control is the most important key for patients’ survival outcomes. Postoperative radiotherapy or chemoradiotherapy should be considered to improve patients’ survival outcomes, especially for the patients with advanced primary site disease or higher cancer stage.

## Figures and Tables

**Table 1:** Clinicopathological and treatment characteristics of patients with buccal mucosa and other oral cancers.

Variables		Buccal	Others	*p* Value
		*n* = 92	*n* = 958	
Clinical characterstics				
Age	Range (Median)	47–94 (73)	21–98 (69)	<0.01
Gender	Male (%)	59 (64%)	591 (61%)	0.63
	Female (%)	33 (36%)	368(39%)	
Alcohol	None/Sometimes (%)	56 (61%)	510 (53%)	0.26
	Habitual (%)	36 (39%)	430 (45%)	
	Unknown (%)	0 (0%)	18 (2%)	
Smoking	Never/Former (%)	58 (63%)	581 (61%)	0.7
	Current (%)	33 (36%)	364 (38%)	
	Unknown (%)	1 (1%)	13(1%)	
UICC TNM classification				
cT classification	Tis/T1 (%)	28 (30%)	283 (30%)	0.01 *
	T2 (%)	42 (46%)	316 (33%)	
	T3 (%)	9 (10%)	138 (14%)	
	T4a (%)	7 (8%)	209 (22%)	
	T4b (%)	6 (7%)	12 (1%)	
cN classification	N0 (%)	66 (72%)	638 (67%)	<0.01 **
	N1 (%)	13 (14%)	113 (12%)	
	N2 (%)	11 (12%)	188 (20%)	
	N3 (%)	2 (2%)	19 (2%)	
M classification	M0 (%)	91 (99%)	953 (99%)	0.49
	M1 (%)	1 (1%)	5 (1%)	
c Stage	I (%)	27 (29%)	267 (28%)	0.20 ***
	II (%)	29 (32%)	236 (25%)	
	III (%)	14 (15%)	135 (14%)	
	IV (%)	22 (24%)	320 (33%)	
Treatment	Surgery (%)	83 (90%)	899(94%)	0.22
	Others (%)	9 (10%)	59 (6%)	

* comparison between Tis/T1/T2 vs. T3/T4, ** comparison between N0 vs. N1/N2/N3, *** comparison between I/II vs. III/IV.

**Table 2:** Characteristics of surgical cases with buccal mucosa and other oral cancers.

**Variables**		Buccal	Others	*p* Value
		*n* = 83	*n* = 899	
Primary Resection				
	Per oral	77 (93%)	739 (82%)	0.01
	External	6 (7%)	160 (18%)	
Neck dissection				
	Yes	30 (36%)	479 (53%)	<0.01
	No	53 (64%)	420 (47%)	
Reconstruction				
	None	19 (23%)	236 (26%)	
	Artifiial material	54 (65%)	478 (53%)	
	Local/pedicled flap	0 (0%)	53 (6%)	
	Free flap	10 (12%)	132 (15%)	
Postoperative treatment				
	Yes	15 (18%)	219 (24%)	0.20
	No	68 (82%)	680 (76%)	
Surgical margin				
	Positive	4 (5%)	71(8%)	0.31
	Negative	79 (95%)	828 (92%)	
Positive nodes				
	pN positive	19 (23%)	214 (24%)	0.85 *
	pN negative	11 (13%)	265 (29%)	
	not dissected	53 (64%)	420 (47%)	
Extracapsular extension				
	Yes	4 (5%)	58 (6%)	0.56 **
	No	15 (18%)	156 (17%)	
	no positive nodes	64(77%)	685(77%)	

* comparison between pN positive vs. pN negative/not dissected, ** comparison between Yes vs. No/no positive noses.

**Table 3:** Survival differences of patients with buccal cancer among various clinicopathological covariates in terms of OS and RFS.

Variables		Overall Survival		*p* Value	Recurrence-Free Survival		*p* Value
		2 Years	5 Years		2 Years	5 Years	
Age							
	>73	82.8%	77.5%	0.24	70.7%	63.4%	0.65
	73≦	87.1%	84.2%		77.6%	65.9%	
Gender							
	Male	88.6%	82.3%	0.70	73.6%	71.4%	0.98
	Female	85.7%	77.4%		75.2%	51.0%	
cT classification							
	Tis/T1/T2	91.0%	87.5%	<0.01	80.4%	72.7%	0.04
	T3/T4	71.4%	48.2%		57.4%	45.9%	
cN classification							
	N0	91.8%	87.9%	0.02	85.0%	75.0%	0.01
	N1/N2/N3	75.0%	59.2%		50.0%	44.4%	
c Stage							
	I/II	94.5%	92.2%	<0.01	85.5%	76.0%	<0.01
	III/IV	76.8%	56.1%		50.6%	40.9%	
Neck dissection							
	Yes	79.7%	72.5%	0.22	66.7%	58.3%	0.50
	No	92.2%	85.5%		82.2%	73.8%	
Postoperative treatment							
	Yes	73.3%	66.7%	0.07	60.0%	32.8%	0.02
	No	90.9%	83.9%		80.2%	75.9%	
pN classification							
	N0	90.3%	86.6%	0.10	83.6%	74.0%	0.03
	N1/N2/N3	78.9%	61.8%		52.6%	46.1%	
Surgical margin							
	Positive	66.6%	66.6%	0.77	66.7%	66.7%	0.98
	Negative	87.1%	81.2%		76.7%	67.7%	
Extracapsular extension							
	Positive	50.0%	50.0%	0.08	50.0%	50.0%	0.22
	Negative/no positive nodes	89.6%	82.2%		77.7%	68.6%	

**Table 4:** Survival differences of patients with buccal cancer among various clinicopathological covariates in terms of OS and RFS.

Variables		Overall Survival				Recurrence-Free Survival			
		HR	95%CI		*p* Value	HR	95%CI		*p* Value
Age	>73 vs. 73≦	0.644	0.207	2.00	0.45	0.804	0.344	1.880	0.61
Gender	Male vs. Female	1.904	0.627	5.77	0.26	1.624	0.708	3.724	0.25
cT classification	T3/T4 vs. Tis/T1/T2	4.921	1.519	15.94	<0.01	3.084	1.001	9.500	0.04
cN classification	N1/N2/N3 vs. N0	2.376	0.359	15.72	0.37	3.474	0.344	22.700	0.19
Neck dissection	Yes vs. No	0.473	0.114	1.95	0.30	0.123	0.021	0.724	0.02
Postoperative treatment	Yes vs. No	1.188	0.284	4.95	0.81	2.121	5.985	5.985	0.15
pN classification	N1/N2/N3 vs. N0	0.944	0.172	5.17	0.95	2.804	0.541	14.520	0.21
Surgical margin	Positive vs. Negative	2.046	0.231	18.07	0.52	0.719	0.093	5.571	0.75
Extracapsular extension	Positive vs. Negative/no positive nodes	1.027	0.151	6.96	0.98	0.344	0.054	2.180	0.25

## Data Availability

The data presented in this study are available on request from the corresponding author.
